# The prevalence of proximal junctional kyphosis (PJK) and proximal junctional failure (PJF) in patients undergoing circumferential minimally invasive surgical (cMIS) correction for adult spinal deformity: long-term 2- to 13-year follow-up

**DOI:** 10.1007/s43390-021-00319-1

**Published:** 2021-03-16

**Authors:** Neel Anand, Aniruddh Agrawal, Robert Ravinsky, Babak Khanderhoo, Sheila Kahwaty, Andrew Chung

**Affiliations:** 1 Department of Orthopaedics, Cedars Sinai Medical Center, 444 S. San Vicente Blvd., #900, Los Angeles, CA 90048 USA; 2 Topiwala National Medical College, Mumbai, India; 3 Department of Orthopaedic Surgery, University of Arizona College of Medicine, Phoenix, AZ USA

**Keywords:** Circumferential minimally invasive surgery (CMIS), Adult spinal deformity (ASD), Proximal junctional kyphosis (PJK), Proximal junctional failure (PJF)

## Abstract

**Objectives:**

This aim of this study is to evaluate the prevalence of PJK and PJF in patients who underwent circumferential minimally invasive surgery (cMIS) for ASD.

**Methods:**

A prospective database of patients who underwent cMIS correction of ASD from November 2006 to July 2018 was queried. PJK was defined as angle > 10° and at least 10° greater than the baseline when measuring UIV to UIV + 2. PJF was defined as any type of symptomatic PJK which required surgery. Pre-op, latest and delta SVA and PI-LL mismatch were compared between patients with PJK and without. Only patients instrumented at 4 or more levels with full length 36″ films and a minimum 2-year follow-up were included.

**Results:**

A total of 184 patients met inclusion criteria for this study. Mean follow-up time was 85.2 months (24–158.9 months, SD 39.1). Mean age was 66 years (22–85 years). The mean number of operated levels was 6.9 levels (4–16 levels, SD 2.8). A total of 21 patients (10.8%) met PJK criteria. Only 10 (4.9%) were symptomatic (PJF) and underwent revision surgery. The other 11 patients remained asymptomatic. Comparing PJK to non-PJK patients, there was no statistically significant difference in the post-op SVA, delta SVA, post-op PI/LL and delta PI/LL between the two groups.

**Conclusion:**

Our study would suggest that in the appropriately selected and well-optimized patient, CMIS deformity correction is associated with a low prevalence of PJK and PJF.

## Introduction

Adult spinal deformity (ASD) is one of the most common and disabling conditions in the elderly population worldwide with a prevalence ranging from 6.0 to 68.0% [1–4]. Conservative treatment can be pursued for mild to moderate degenerative scoliosis as patients may be asymptomatic, and it is rare for these conditions to progress. However, indications for surgical intervention have been very well established in the literature and include failure of pain control by conservative treatment and unappealing cosmetic appearance [5–7].

The surgical treatment of this condition necessitates a complex, multi-level approach, frequently requiring osteotomies, and occasionally a combination of anterior and posterior approaches. The surgical time, length of hospital stay, blood loss, length of recovery and extent of soft tissue dissection are usually far greater than those associated with procedures for common degenerative conditions of the spine [8–10]. This results in a greater frequency of intra-operative and post-operative complications, the prevalence of which range between 13 and 39%. [11, 12]. One such increasingly recognized complication of long-segment instrumentation for correction of kyphosis and scoliosis is proximal junctional kyphosis (PJK), which can progress to proximal junctional failure (PJF) [1–6].

Although the definition of PJK and PJF varies throughout the literature, PJK has traditionally been defined as a final proximal junctional sagittal Cobb angle greater than 10 degrees and a postoperative angle at least 10 degrees greater than the preoperative value, as measured between the lower endplate of the upper instrumented vertebrae (UIV) and the upper endplate of the second supra-adjacent vertebrae (UIV + 2) [13]. The cause of this complication is multi-factorial and includes progressive deformity from aging, disruption of the posterior ligamentous complex (facet capsules, ligamentum flavum, interspinous ligament and supraspinous ligament), vertebral fracture and low bone density, instrumentation failure and degenerative disk disease [13–16]. While various sources cite a PJK rate ranging from 5 to 46% [17–30] for open spinal deformity procedures, most studies cite a rate of approximately 20–40%.

Some risk factors for PJK have been established and include age > 55 years, fusion to the sacrum, combined anterior and posterior surgery, thoracoplasty and instrumentation of T1–T3 vertebrae [13, 14, 19, 31–33]. It has also been theorized that use of minimally invasive (MIS) posterior pedicle screw instrumentation limits dissection of the posterior muscular structures/tension band and may decrease the incidence of PJK compared with open pedicle screw placement; however, this has not yet been conclusively demonstrated [34]. In fact, Mummaneni et al. previously demonstrated an incidence rate of 31.3% for occurrence of PJK in MIS posterior adult deformity correction [34]. This was a retrospective multi-centre study where patients had interbody fusions with open posterior pedicle screws (hybrid) or percutaneous pedicle screws for adult spinal deformity. It was early in the experience of most centres, far fewer levels were fused with MIS technique compared to hybrid, no specific protocol was in place for MIS patient selection, and no techniques for minimally invasive posterior rod reduction, derotation or translation were used. There was a trend, when propensity matched for the number of levels fused, toward less PJK in the percutaneous group when compared to the open pedicle screw group.

Anand et al. have published on a their circumferential minimally invasive surgery (cMIS) protocol for ASD correction and have demonstrated significant improvements in both sagittal and coronal alignment, and patient-reported functional scores with comparatively lower rates of morbidity when compared to open techniques [35, 36]. However, the prevalence of PJK and PJF in patients undergoing cMIS deformity correction has not been defined. The aim of this study is therefore to determine the prevalence of PJK and PJF in ASD patients having undergone cMIS techniques utilizing a staged protocol in a single centre.

## Materials and methods

A single-centre consecutive series from a prospectively collected database was queried for all patients who had undergone cMIS correction of ASD (Cobb > 20 or SVA > 50 mm or PI/LL mismatch > 10) at 4 or more levels between November 2006 and July 2018. This yielded 213 patients. Those without a full-length radiograph or 2-year follow-up were excluded. All patients had severe back pain with or without leg pain that was non-responsive to at least 6 months of conservative therapies secondary to adult spinal deformity. All patients underwent cMIS correction of adult spinal deformity without any posterior osteotomy of any kind.

The staged approach has evolved over the past 13 years, and from 2011, we have had a consistent approach [35] which has previously been described in great detail [35, 36]. The “old” protocol was as follows: During stage 1, multilevel lateral lumbar interbody fusions (LLIFs) were performed. During stage 2, AxiaLIF was performed at L5-S1 as needed, with subsequent MIS posterior pedicle screw fixation with rod contouring, sequential rod reduction and derotation/translation. The new protocol was instituted in May 2011 and was as such: (Stage 1) multilevel oblique LLIF and MIS L5-S1 oblique interbody fusion (OLIF) or anterior lumbar interbody fusion (ALIF); (Stage 2) MIS pedicle screws with rod contouring, sequential rod reduction and derotation/translation.

Between stages (this includes the old protocol), patients are ambulated. A full-length standing radiograph is obtained on the second day following surgery, and spino-pelvic parameters are reassessed. Any persistence of radicular pain or neurogenic claudication is also noted during this time. These data are then utilized to plan for the second stage accordingly including the need for additional posterior decompression if needed. Only four patients have needed a posterior microdecompression due to persistent radiculopathy post-stage 1.

On post-operative day 3, the second stage is performed with posterior percutaneous pedicle screw instrumentation. For both protocols, 5.5-mm titanium alloy rods were utilized. Posterior segmental “pars-facet-pars” fusions were done at those segments that did not have an interbody fusion. These were usually proximal to the L1-2 segment. No patient had any posterior column osteotomy or an ACR procedure other than at L5-S1 where an ALIF would require release of the ALL.

Preoperative and postoperative radiographic parameters, level of UIV, age and duration of follow-up were analysed. Patient functional outcomes and complication rates were not included in this study as they have already been reported in the articles reporting the surgical technique [35–37]. This study was approved by the institutional review board (IRB).

### Radiographs and measurement of various parameters

On lateral 36-inch X-rays, the sagittal vertical axis (SVA) was measured as the offset from the C7 plumb line to the posterior–superior endplate of S1. Lumbar lordosis (LL) was measured from the upper endplate of L1 to the upper endplate of S1. Pelvic incidence (PI) was measured as the angle subtended by a perpendicular line from the midpoint of the cephalad endplate of S1 and a line extended to the midpoint of a line connecting the centre of the femoral heads. The LL-PI mismatch was then calculated by subtracting the value of LL from that of the PI (PI-LL mismatch). Pelvic tilt (PT) was defined by the angle subtended by a line connecting the midpoint of the S1 endplate to the vertical plane from the axis centre of the femoral heads.

Furthermore, anatomical and morphological parameters were critically analysed by studying magnetic resonance imaging (MRI) for vascular anatomy, spinal stenosis and disc degeneration. Computed tomography (CT) was used to assess for segmental fusions and vacuum sign on discs. All patients underwent dual-energy X-ray absorptiometry (DEXA) scans to determine whether anabolic agents were required to augment bone density.

### Criteria for proximal junctional kyphosis and proximal junctional failure

PJK was defined as angle > 10° and at least 10° greater than the baseline when measuring the lower endplate of the UIV and the upper endplate of 2 vertebrae supra-adjacent (UIV + 2). PJF was defined as any type of symptomatic PJK requiring surgery, including fracture of the vertebral body of UIV or UIV + 1, implant failure at the UIV (failure of UIV fixation), or herniation of the disk immediately cranial to the UIV.

### Analysis

In addition to calculating the prevalence of PJK in those patients meeting inclusion criteria, preoperative, latest post-operative, delta SVA and PI-LL mismatch were compared between patients with PJK and without. Mean and standard deviation were used to describe continuous variables. Comparisons between patients that developed PJK/PJF and those that did not were also performed. *T* tests and Chi-squared analysis were used for continuous and categorical variables, respectively. Studies were two-sided, and *p* < 0**.**05 was considered statistically significant. All statistical analyses were conducted using SPSS (version 21).

## Results

A total of 184 patients met inclusion criteria. The mean age was 66 years (22–85 years). There were 106 females and 78 males. Mean follow-up was 85.2 months (24–158.9 months). Mean operated levels were 6.9 levels (4–16 levels, SD 2.8). UIV levels are shown in Table 1. In 114 patients (65.8%), the UIV was between T10 and T12.

**Table 1:** Demographic data

Cohort parameters	Value derived
Age	Average = 66.1 years (22–85 years)
Number of operated levels	Average = 6.7 levels (3–16 levels)
Level of upper instrumented vertebrae	T2–T4: 18 cases (9.8%)
	T9: 4 cases (2.2%)
	T10: 54 cases (29.3%)
	T11: 22 cases (11.9%)
	T12: 38 cases (20.6%)
	L1: 27 cases (14.7%)
	L2: 21 cases (11.4%)

A total of 21 patients (11.4%) met PJK criteria. Only 10 (2.7%) of these were symptomatic (PJF) and underwent revision surgery. Revision surgery was done for these patients at various intervals post-surgery ranging from 5 to 60 months. Table 2 highlights information about the post-operative duration after which the repeat surgery was performed for patients suffering from PJF. Table 3 demonstrates the relative severity of deformities (PJK versus no PJK) based on the Schwab classification. Table 4 describes the extent of fusion with all deformities.

**Table 2:** Patients that suffered from PJK and Time to Revision Surgery

Outcome measure	Number of patients (percentage)
Total patients that met PJK criteria	21 patients (11.4%)
Patients that after PJK, met PJF criteria and needed re-surgery	10 patients (5.4%)
Timing for revision surgery	
(1) Within 6 months	1 patient (10% of PJF cohort)
(2) Between 6 and 12 months	1 patients (10% of PJF cohort)
(3) Within 12 and 24 months	4 patients (40% of PJF cohort)
(4) Within 24 and 72 months	4 patients (40% of PJF cohort)

**Table 3:** Curve types and deformity severity (PJK versus No PJK) based on SRS-Schwab classification

	All (*n* = 184)	PJK group (*n* = 21)	No PJK group (*n* = 163)	
Double	12	1	11	
Thoracic	2	0	2	
Thoracolumbar/lumbar	114	15	99	
No major coronal deformity	56	5	51	

SRS-schwab sagittal modifiers				
	All	PJK	No PJK	*P* value
PT				
0	60	6	54	*P* > 0.05
+	79	7	72	*P* > 0.05
+ +	45	8	37	*P* > 0.05
SVA				
0	57	4	53	*P* > 0.05
+	85	13	72	*P* > 0.05
+ +	42	4	38	*P* > 0.05
PL-LL				
0	58	3	55	*P* > 0.05
+	73	8	65	*P* < 0.05
+ +	53	10	43	*P* > 0.05

*P*-values reflect comparisons between PJK vs no PJK

**Table 4:** Extent of fusion

	All (*n* = 184)	PJK (*n* = 21)	No PJK (*n* = 163)
Lower-instrumented vertebral level			
LIV above sacrum	41	3	38
Extension of fusion to sacrum	143	18	125
Upper-instrumented vertebral level			
UIV T2-4	18	2	16
UIV T9	4	1	3
UIV T10	54	7	47
UIV T11	22	5	17
UIV T12	38	3	35
UIV L1	27	3	24
UIV L2	21	1	20

There were no catastrophic failures other than one patient with a compression fracture. All patients with PJF experienced a gradual loss of sagittal alignment secondary to aging and pelvic decompensation rather than a catastrophic failure. The 11 patients (64.3% of PJK cohort) that did not meet criteria for PJF but met criteria for PJK remained asymptomatic and did not need revision surgery.

Table 5 showcases the different pre-operative parameters between the group that suffered from PJK and those that did not suffer from PJK. Table 6 shows that there was no statistically significant differences in the post-op coronal Cobb, SVA, PI/LL mismatch and PT between the two groups.

**Table 5:** Pre-op radiographic data pre-op

	PJK	Non-PJK	P value
Coronal Cobb	35.1 (11.3–61.8) SD 13.7	33.8 (15–74.7) SD13.9	*P* > *0.05*
SVA	65.8 (9.6–123.4) SD 34.9	54.1 (3.3–223) SD 44.8	*P* > *0.05*
PI/LL mismatch	18.8 (1.7–49.2) 12.5	18.1 (0.3–60.9) SD 14.7	*P* > *0.05*
PT	26.2 (15–39.2 8.7	24.7 (1.7–53) SD 9.9	*P* > *0.05*

*SVA* sagittal vertical axis, *PI* pelvic incidence, *LL* lumbar lordosis, *PT* pelvic tilt

**Table 6:** Post-op radiographic data

	PJK	Non-PJK	*P* value
Coronal Cobb	12.5 (1.6–31.3) SD 7.7	12.59 (0–46.2) SD 9.2	*P* > *0.05*
SVA	43 (4.8–146.3) SD 37.7	36.8 (0–127) SD 29.4	*P* > *0.05*
Delta SVA	37.9 (4.8–96.6) SD 28.9	31.2 (0.5–186.4) SD 29.7	*P* > *0.05*
PI/LL mismatch	11.2 (06–32.5) 8.5	10.3 (0.1–33) SD 6.9	*P* > *0.05*
PT	27.4 (4–37.2) 7.9	25 (5.8–53) SD 8.8	*P* > *0.05*

*SVA* sagittal vertical axis, *PI* pelvic incidence, *LL* lumbar lordosis, *PT* pelvic tilt

## Discussion

The rates of PJK after open deformity correction have been typically quoted as ranging from 20–40%. Over the past five to ten years, a substantial amount of research has been devoted to the study PJK and PJF, and a number of both modifiable and non-modifiable risk factors for PJK/PJF have been elucidated [13, 14, 18, 19, 31, 33]. Our rates of PJK and PJF were 7.6 and 2.7%, respectively, for a consecutive cohort of patients with ASD undergoing staged cMIS deformity correction. These numbers do contrast starkly with the rates noted above for open deformity surgery. By its very design, our staged cMIS protocol has attempted to address and optimize several of the modifiable risk factors for PJK/PJF, while enabling adult spinal deformity correction and minimizing complications [35].

Preservation of the posterior midline tension band has often been proposed as an important technique by proponents of MIS deformity surgery. While the literature supports the notion that sparing of the facet capsules and midline ligamentous structures via the use of percutaneous posterior fixation is beneficial with respect to reducing the rates of PJK/PJF [13, 14, 17, 20, 24, 29–31, 38–42], we feel that this alone does not fully account for the results observed in our study. For instance, Mummaneni et al. did not observe lower rates of PJK at a statistically significant level when employing percutaneous posterior instrumentation in comparison with open surgery [34].

Several authors have observed that osteoporosis is a significant risk factor for PJK [17, 43–49]. With the reduction of PJK rates in mind, the process of optimizing patients for cMIS deformity correction surgery begins with an appropriate investigation of bone density via DEXA scanning. All patients in this study were managed based on their *T*-scores within 6 months prior to surgery. If a patients *T*-score was less than − 3.0, MIS surgery was contraindicated and patients were started on Teriparatide for a minimum of three months before considering surgery again. If *T*-score was between − 2.0 and − 3.0, patient was indicated for surgery and daily injection of Teriparatide (Forteo) and was recommended and continued for 1-year post-surgery. To date, we have only had one proximal osteoporotic fracture and one UIV end plate fracture which suggests that treating osteoporosis is an important part of the overall strategy of surgically managing adult spinal deformity [47].

Another important preoperative factor is the selection of an appropriate UIV. A number of authors have cited stopping the construct cranially at a kyphotic or degenerated segment as a risk factor for PJK [20]. In our cohort, the selection of the UIV was done by choosing the lowest possible neutral vertebrae with a well preserved intervertebral disk above. Hence as per our protocol all vertebrae in the Cobb angle were included in the fusion. The upper-instrumented level was the first normal parallel disc on X-ray and MRI. L5-S1 was included if there was any of the following including stenosis, spondylolisthesis, obliquity, degeneration, sagittal imbalance greater the 10 cm, or osteoporosis [35]. Ultimately, we did not find that UIV selection meaningfully affected the prevalence of PJK in our patient population.

Another frequently cited risk factor for PJK is the use combined anterior/posterior fusions [21, 24, 31]. In general, while the cMIS protocol involves staged anterior interbody fusions followed by percutaneous posterior pedicle screw fixation, when extending into the thoracic spine, circumferential fusion is not performed at the UIV. Of the 184 patients included in this study, the majority (136/184) were fused into the thoracic spine (Table 2), where the cranial-most LLIF during the anterior stage 1 procedure was performed at L1-2. Hence as an example, in a T10 to pelvis construct, all the lumbar levels from L1-2 to L5-S1 had an interbody fusion, whereas the cranial portion of the fusion construct from T10 to L1 involves a posterior-only instrumentation and fusion. This helps reduce the gradient of stiffness of the proximal construct and thereby reduce the stresses at the proximal junctional level.

Furthermore, with respect to the posterior fusion at the cranial portion of the construct, we respect the posterior midline structures and defer an extensive midline stripping and posterolateral dissection. Alternatively, a minimally invasive decortication and grafting of the pars/facet/pars complex is performed at each level that does not have an interbody fusion, through the percutaneous screw incisions, thus minimizing soft tissue disruption.

Another often quoted reason for PJK is related to the stiffness of materials in the construct, and the significant forces imparted by a stiff construct above the UIV [40, 41]. Our percutaneous screw-rod reduction techniques involve sequential translation and derotation manoeuvres over a well contoured rod. As reduction towers are deployed and the spine is reduced to the rod, the very nature of the reduction depends on a certain give and take between the screw–bone interface and the rod itself. A stiff cobalt-chrome rod we strongly advice against using in cMIS techniques as it transfers significant stress to the screw–bone interface and results in pull out of the screw. Hence all patients in our cohort had 5.5-mm titanium alloy rods, while deferring the use of rods with larger diameters, or those with a higher stiffness. This again helps reduce the gradient of stiffness across the proximal junction and thereby helping to prevent subsequent PJF.

A rod of lesser diameter or reduced stiffness had been linked with increasing rates of rod fractures [50, 51]. Our rod fracture rate in the cohort was 2.7% (5 patients). 4 other patients had loosening of the nut at the iliac screw. All these patients went on to have solid L5-S1 fusion on CT scan and have not had a revision. This failure rate again is low compared to published reports of rod failure with open surgery [50–60]. This may be due to two reasons. Firstly, there is load sharing within the construct due to interbody fusion at the lumbar levels. Secondly, the rod is contoured strictly only in the sagittal plane with no coronal bends or in situ bending manoeuvres. This respects the notch sensitivity of titanium and reduces the areas of stress induced rod failure. The only area of in situ bending if any is at the distal end of the rod to bend the rod down into the iliac screw which occurred in the 5 cases of distal rod failure in our cohort.

Lastly, fusion to the sacrum or pelvis has been frequently cited as a risk factor for PJK [17, 19, 24, 61]. While extension of the construct to the sacrum/pelvis is often unavoidable due to the presence of significant degeneration, or instability, we could not show any difference in our patients with PJK and those without PJK.

Previous studies have noted that greater sagittal imbalance may be a risk factor for PJK [62], however, we did not find this to be the case in our patient population. Further, while other studies found that increased sagittal correction, or delta SVA, could predispose patients to PJK, the magnitude of correction was not observed to be a significant contributing factor in our study. Similarly, the delta PI-LL mismatch was not significantly different between cohorts, again suggesting that the magnitude of deformity correction in the sagittal plane did not play a significant role in the development of PJK in our patient population.

### What is the clinical impact of our findings?

While a number of technical measures have previously been suggested that may reduce rates of PJK, most of these measures have demonstrated limited success [62]. PJK/PJF continues to be a concerning source of morbidity and disability for the patient, a challenging problem for the spine surgeon, and an unwanted financial expenditure for the payers. Our data suggests that a thorough preoperative evaluation and risk mitigation strategy combined with a minimally invasive, staged surgical approach may help to reduce perioperative complications and are associated with a low prevalence of PJK and PJF. The other stark difference we have seen in our series is the nature of PJK/PJF. There were no catastrophic PJFs but rather a gradual decompensation and loss of sagittal alignment over years. This is likely the result of a gradual loss of pelvic compensation and progressive thoracic kyphosis as patients age.

### What are the limitations of this study?

This study is not without limitations. First, this study represents a case series without a comparator group of patients undergoing open or hybrid deformity corrections. The lack of an appropriate comparator group raises questions about random variation within the population, and the possibility that these results were observed due to random chance. However, despite the absence of a randomized, or alternatively a propensity-matched comparator group, the size of the cohort allows us to confidently draw conclusions about the impact of the cMIS protocol on PJK and PJF rates. That being said, whether these techniques definitively reduce the prevalence of PJK or PJF compared to open techniques would of course require a matched, controlled study. Another limitation of this study comes from the fact that this cohort represents a series of patients treated by a single surgeon, of considerable experience, at one institution. This fact raises issues of generalizability of results to other populations in the hands of less experienced surgeons. However, the cMIS protocol, including preoperative management as well as a detailed technical description of the procedures, has been previously published. This protocol can be learned and adopted with success in a variety of hospital environments and healthcare systems.

Despite the aforementioned limitations, this study does have significant strengths. These strengths include a medium-long term follow-up (mean follow up of 85.2 months), and the utilization of prospectively collected data which eliminates both hindsight and recall bias.

## Conclusions

Our study would suggest that in the appropriately selected and well-optimized patient, CMIS deformity correction is associated with a low prevalence of PJK and PJF. Prospective comparative studies would be helpful in determining whether these techniques definitively reduce the prevalence of PJK or PJF when compared to open techniques.
